# Monomeric, Oligomeric and Polymeric Proteins in Huntington Disease and Other Diseases of Polyglutamine Expansion

**DOI:** 10.3390/brainsci4010091

**Published:** 2014-03-03

**Authors:** Guylaine Hoffner, Philippe Djian

**Affiliations:** Génétique moléculaire et défense antivirale, Centre National de la Recherche Scientifique, Université Paris Descartes, 45 rue des Saints Pères, 75006 Paris, France

**Keywords:** huntington disease, huntingtin, protein aggregation, neuronal inclusions, oligomers, fibrils, protein secondary structure, amyloid, β-sheets, neurodegenerative diseases

## Abstract

Huntington disease and other diseases of polyglutamine expansion are each caused by a different protein bearing an excessively long polyglutamine sequence and are associated with neuronal death. Although these diseases affect largely different brain regions, they all share a number of characteristics, and, therefore, are likely to possess a common mechanism. In all of the diseases, the causative protein is proteolyzed, becomes abnormally folded and accumulates in oligomers and larger aggregates. The aggregated and possibly the monomeric expanded polyglutamine are likely to play a critical role in the pathogenesis and there is increasing evidence that the secondary structure of the protein influences its toxicity. We describe here, with special attention to huntingtin, the mechanisms of polyglutamine aggregation and the modulation of aggregation by the sequences flanking the polyglutamine. We give a comprehensive picture of the characteristics of monomeric and aggregated polyglutamine, including morphology, composition, seeding ability, secondary structure, and toxicity. The structural heterogeneity of aggregated polyglutamine may explain why polyglutamine-containing aggregates could paradoxically be either toxic or neuroprotective.

## 1. Introduction

There are nine diseases of the human central nervous system, each associated with a different protein containing an expanded polyglutamine (polyQ) sequence: spinobulbar muscular atrophy (SBMA) [1], Huntington disease [2], dentatorubral-pallidoluysian atrophy (DRPLA) [3], and spinocerebellar ataxias (SCAs) types 1 [4], 2 [5,6,7], 3 [8], 6 [9], and 7 [10]. A TATA-binding protein containing an expanded polyQ is the cause of the rare SCA17 [11]. In the causative proteins of all nine diseases, there is normally a polyQ of about 20 glutamine residues. When the number of glutamines exceeds a value of about 35, the protein produces disease of the central nervous system [12]. The most frequent of these diseases is Huntington disease: there are about 25,000 cases and 100,000 persons at risk in the United States. Huntington disease associates abnormal movements and a progressive cognitive impairment leading to dementia [13]. The disease affects predominantly the striatum and the cerebral cortex [14,15]. Although most forms of the disease start in adulthood, there exist juvenile forms, which are associated with polyQ expansions longer than 65 residues [16].

The nine diseases of polyQ expansion share a number of properties: (1) autosomal dominant inheritance, with the exception of SBMA, which is linked to the X chromosome; (2) anticipation, which means that the disease tends to begin earlier and to become more severe with successive generations in affected families, generally when the expanded allele is transmitted by the father; (3) close correlation of disease severity with polyQ length; (4) commonly late onset: for instance, the mean age of onset for Huntington disease is 38 years; and (5) all the diseases affect the central nervous system, although the proteins are generally present in similar amounts in a large variety of unaffected tissues. The fact that the diseases of expanded polyQ share so many specific properties argues strongly for a common pathogenic mechanism.

Many arguments support a toxic gain of function by the expanded protein [17]. It appears very likely that the polyQ sequence in itself, independently of its protein context, is toxic for neurons. The toxicity of the expanded protein is presumably related to the generation by proteolysis of peptides bearing the expanded polyQ. These protein fragments become misfolded and acquire aggregating properties [18,19,20,21].

This review focuses on polyQ aggregation and the characteristics of inclusions, fibrils, oligomers, and monomers of polyQ. A comprehensive view of the mechanisms of formation, and of the secondary structure and toxicity of aggregated and unaggregated polyQ species is given. Huntingtin is examined with particular attention as it is associated with the most frequent and the most investigated of the neurological diseases caused by polyQ expansion.

## 2. General Mechanism of PolyQ Aggregation: Formation of β-Sheet Rich Aggregates

### 2.1. Discovery of PolyQ Aggregation

Poly-l-glutamine is insoluble in water. Modeling has suggested that polyQ could form polar zippers made of antiparallel β strands linked together by hydrogen bonds between main-chain and side-chain amides [22]. The first evidence for polar zipper formation by polyQ sequences came from the discovery that a synthetic peptide with the sequence R_2_Q_15_K_2_ aggregates into pleated β-sheets. At neutral pH, the peptide forms worm-like particles with an X-ray diffraction pattern typical of cross-β type fibers [23]. Incorporation of Q_10_ into a small protein results in the formation of oligomers and abnormal monomers whose glutamine repeats are presumably bent into hairpins. It was proposed that proteins bearing an expanded polyQ were toxic because they acquired an excessive affinity for each other or for other proteins with a polyQ sequence [24]. Perutz later hypothesized that poly-l-glutamine fibers consist of amyloid β-helix tubes. A single helical turn with 20 residues would be unstable but two turns with a total of 40 residues would be held together by hydrogen bonds between the amides of successive turns and could act as a nucleus for further helical growth. This would explain why the pathological threshold of most diseases of polyQ expansion is about 40 glutamines [25].

R6/2 transgenic mice expressing the first exon of human huntingtin, which encodes about 130 glutamine residues flanked by 17 *N*-terminal and 52 *C*-terminal residues develop neurological disease [26] and neuronal inclusions [27]. The bacterially produced protein encoded by the exon-1 of huntingtin forms aggregates *in vitro* when its polyglutamine sequence exceeds 51 residues, but not when the polyQ is 20 or 30 residues. As purified bacterial peptide was used in these experiments, aggregation must have resulted from multimerization by polar zipper formation [28]. The authors concluded that the inclusions of exon-1 transgenic mice reported by Davies and colleagues [27] were similarly stabilized by hydrogen-bonded polar zippers, but there was no direct analysis of the intermolecular bonds found in the inclusions of mice. Microscopic inclusions containing the protein with expanded polyglutamine are present in the neurons of the brain regions affected by each disease of polyglutamine expansion [21,29,30,31,32,33,34,35,36].

The use of concentrated formic acid, which is an extremely effective solvent for otherwise insoluble proteins, provided the first evidence of the noncovalent aggregation of polyQ, presumably by formation of polar zippers. Hazeki and coworkers showed that COS cells expressing the exon-1 of huntingtin, encoding an expanded polyQ, accumulate large aggregates, which can be reduced to monomer by concentrated formic acid [37]. It was later shown that the perinuclear inclusions formed in PC12 cells by an expanded polyQ flanked by the residues adjacent to the polyQ of the androgen receptor (associated with spinobulbar muscular atrophy) are mostly reduced to monomer after treatment with 96% formic acid [38]. Use of concentrated formic acid has also provided indirect evidence as to the existence of polar zippers in transgenic mice and in the brain of patients with Huntington disease [20,39].

### 2.2. Do Aggregates of PolyQ Possess An Amyloid Structure?

A vast array of techniques has been used to investigate the structure of aggregated polyQ and it has been established that polyQ aggregates are rich in β-sheets. As polyQ aggregates often appear as filaments, it has been concluded that they are amyloid or amyloid-like. Amyloid fibrils are strictly defined as filamentous aggregates characterized by the presence of cross-β structural motifs with specific secondary, tertiary, and quaternary structures, a definition derived from X-ray and electron diffraction studies [40,41,42,43,44]. Although polyQ aggregates meet a number of the criteria that define amyloid fibrils, it is still unclear whether they are strictly speaking amyloid. The arguments for the amyloid nature of polyQ aggregates are as follows:

It has been found by various means that polyQ aggregates are rich in β-sheets [23,45,46,47].

Aggregates of polyQ formed *in vitro* bind both thioflavin T and Congo red [28,47,48], two stains widely used in the diagnosis of amyloidosis.

Electron microscopy has revealed that polyQ forms aggregates resembling amyloid fibrils in all respects [45,47].

PolyQ aggregates possess an epitope associated with the amyloid-folding motif [47].

The amyloid nature of the inclusions formed in the brain of both transgenic mice and patients has been questioned as most of these inclusions are not stained by Congo red [49,50]. However, when the SDS-resistant aggregates found in Huntington disease brain are collected on cellulose acetate filters, multiple Congo red positive particles are detected [48]. It seems likely that the SDS removes from the inclusions proteins that are co-aggregated with the polyQ-bearing protein (see, for example, [51,52,53]) and that prevent Congo red binding. Some experimental evidence has also suggested that polyQ aggregates are stabilized by covalent bonds catalyzed by transglutaminases [54]. It appears likely that the polyglutamine initially multimerizes into Congo red-binding amyloid fibrils. The amyloid fibrils then coaggregate covalently or not with other proteins to form the inclusions, which can no longer bind Congo red.

### 2.3. Are PolyQ β-Sheets Parallel, Antiparallel, or Both?

In amyloid fibrils the stacks of β-sheets are generally stabilized by hydrophobic interactions and in-register parallel β-sheets constitute the most common arrangement of the β-strands. In the case of polyQ, hydrophobic interactions are replaced by hydrogen bonds between the side chains of the glutamine residues. Such bonding is compatible with either a parallel or an antiparallel arrangement of the β-strands.

Perutz and colleagues initially proposed a model in which a peptide with Q_15_ adopts an antiparallel configuration of the β-sheets [23]. Perutz later proposed a new model in which poly-l-glutamine longer than 40 residues forms amyloid β-helix tubes with parallel β-strands [25]. These studies raise the question of a structural difference between polyQs below and above pathological length. However, most experimental investigations of polyQ β-strands favor an antiparallel arrangement whether the polyQ is below or above pathological length. Studies by electron paramagnetic spectroscopy of the aggregated huntingtin exon-1 fragment are inconsistent with in-register parallel β-sheets, and suggest instead either loosely arranged parallel structures or an antiparallel β-sheet disposition [55]. Studies by circular dichroism and infrared spectroscopy demonstrate that myoglobin, into which Q_50_ is inserted, aggregates and forms intermolecular antiparallel β-pleated sheets [45]. As demonstrated by solid-state nuclear magnetic resonance spectroscopy, aggregation into fibrils of GK_2_Q_38_K_2_ results in the formation of superpleated β-sheets with individual molecules contributing β-strands to more than one sheet and an antiparallel assembly of strands within β-sheets [56]. In this model, the water filled β-helical structures (β-strands with 20 residues per helical turn) proposed by Perutz and colleagues are disfavored [25]. The huntingtin exon-1 fragment with Q_30_ aggregates into a rigid dehydrated amyloid core, which presumably contains antiparallel β-sheets [57]. X-ray diffraction studies of polyQ homopolymers also support an antiparallel arrangement of the β-sheets [58]. However there is one experimental report favoring parallel β-sheets. From the study by infrared spectroscopy of the aggregation of ataxin-3 (associated with SCA3) with Q_78_, it has been concluded that formation of fibrils is accompanied by a large increase in the amount of β-sheet. The lack of any apparent shoulder in the 1680–1690 cm^−1^ range suggested that the β-strands are parallel [46].

It is possible that polyQ aggregates contain parallel and antiparallel β-sheets. Aggregation of atrophin with Q_56_ in cultured cells has been studied by FRET. In the presence of inclusions, FRET signals are detected whether donor and acceptor fluorescent proteins are each attached to the same side or to the opposite sides of the polyQ repeat. This indicates the existence of parallel and antiparallel β-sheets [59]. The study by synchrotron infrared microspectroscopy of the inclusions found in the brain of patients with Huntington disease has also provided evidence as to the presence of both parallel and antiparallel β-sheets [60]. As these studies were carried out on the bulk of inclusions, it was not possible to know whether the parallel and antiparallel arrangements coexisted in the same inclusions or whether some inclusions contained pure antiparallel β-sheets while others contained parallel β-sheets.

### 2.4. Nucleated Growth Polymerization

The late onset of diseases of polyQ expansion could be attributed to cumulative cell damage. An alternative explanation would be that neuronal death occurs randomly in time, as in radioactive decay, and therefore that the rate of neuronal death is constant in time. Neurons would then have a characteristic half-life, which would be inversely proportional to the length of the polyQ. Neuronal loss in Huntington disease can be evaluated indirectly by measuring ^18^F-doxyglucose uptake in the caudate nucleus [61]. Such measures have shown that the probability of neuronal death in Huntington disease is constant with time [62]. Nucleation of ordered molecular aggregates occurs randomly and could occur rarely within a neuron, but once nucleated, the aggregate would grow rapidly [63]. The study of the kinetics of *in vitro* aggregation of polyQ peptides with Q_28_, Q_36_, Q_42_, and Q_47_ has confirmed that polyQ aggregation proceeds by nucleated growth polymerization. The aggregation nucleus is thought to be a monomer that underwent a transition from a statistical coil to a compact state, presumably rich in β-sheets. This implies that the rate limiting nucleation step in Q_28_, Q_36_, Q_42_, and Q_47_ peptides is the folding of the monomer [64]. However, the aggregation kinetics of a Q_23_ peptide is compatible with a critical nucleus of four molecules, not one. The steady state concentration of the tetrameric nucleus is calculated to be much lower than that of the monomeric nucleus. A decrease in the nucleus size with longer polyQs might explain why the probability of polyQ aggregation is repeat-length dependent [65]. It is worth noting that the monomeric nucleus exists in vanishingly small concentrations and therefore cannot be in itself toxic to cells. Nucleated growth implies that the aggregates are the toxic species and differs from models in which monomeric misfolded forms of expanded polyQ are the toxic species.

### 2.5. Seeded Polymerization

We have seen that polyQ aggregation might require a slow nucleation step followed by rapid polymerization. Once formed, a fibril could act as a seed for its elongation by incorporating either further polyQs (self-seeding) or other proteins (cross-seeding). The RNA-binding protein TIA-1 is sequestered in the inclusions of R6/2 mice and it has been proposed that the polyQ fibrils observed in these mice serve as seeds for the fibrillation of TIA-1 through a *C*-terminal Q/N-rich domain of TIA-1. Cross-seeded fibrils of Q/N-rich proteins might then recruit other proteins that cannot be seeded directly on polyQ fibrils [66].

The ability of insoluble brain proteins of mice bearing a YAC encoding full-length huntingtin with Q_128_ to act as seeds for the polymerization of K_2_Q_44_K_2_ has been studied as a function of age. The insoluble proteins promote polymerization starting at twelve weeks of age, just when motor symptoms begin to appear and long before microscopic inclusions are detected. The seeds likely consist of oligomers of the misfolded expanded huntingtin. Seeding specificity is demonstrated by the fact that neither extracts of Alzheimer’s disease brain, nor prions, can act as seeds for the polymerization of K_2_Q_44_K_2_ [67].

In *Drosophila* transiently expressing an expanded ataxin-3, toxicity of the protein increases with age. The amyloids that form in older flies are more effective at seeding polymerization of recombinant exon-1 huntingtin with Q_62_ than those of younger flies. This suggests that the conformation of amyloids varies with age. Increased toxicity of older amyloids may explain the late onset of diseases of polyQ expansion [68]. Age-related differences of amyloids have been attributed to progressive impairment of intracellular proteolysis due to age-related changes in chaperone levels and chaperone types [69]. The ubiquitin-proteasome system and autophagy have also been incriminated [68,70,71]. The increased toxicity of older amyloids observed in mice and *Drosophila* is in contradiction with the constant rate of neuronal death observed in patients with Huntington disease [62].

## 3. Influence of the Sequences Flanking the PolyQ on Aggregation?

In Perutz’s model of polyQ aggregation by formation of polar zippers, the sequences outside the polyQ are largely irrelevant to the aggregation process. It has been proposed that the antiparallel β-pleated sheets of nonpathological polyQ are not exposed to the solvent, but when the polyglutamine reaches pathological length, it protrudes on the protein surface, becomes accessible to other molecules, and starts aggregate formation. It has been shown indeed that Q_50_ inserted in sperm whale hemoglobin causes partial unfolding of the protein surface without affecting the core [45].

Alternatively, the sequences outside the polyQ may play an important role in aggregation. Expanded polyQ could destabilize the protein and thus expose hydrophobic residues that would then initiate aggregation [72]. Such a mechanism would be similar to that of the formation of amyloid fibrils by lysozyme [73]. However, because the polyQ in itself possesses adhesive properties, there is theoretically no need for residues outside the polyQ to participate in aggregation.

### 3.1. PolyQ Can Cause Neurological Disease Independently of Its Protein Context

Proteins with an expanded polyQ are generally widely distributed and yet each protein affects a distinct neuronal population. This selectivity has been attributed to the influence of the amino acid residues that flank the expanded polyQ. For instance, it has been demonstrated that the protein sequence surrounding the polyQ of ataxin-3 specifies the constituents of nuclear inclusions in cultured cells [74]. However, the expanded polyQ in itself, independently of its protein context, is toxic for neurons. Q_146_ inserted in the enzyme hypoxanthine phosphoribosyl transferase, a protein totally unrelated to any of the disease-causing proteins, produces neuronal inclusions and disease of the nervous system in mice [75]. A transgene encoding an expanded polyQ surrounded by only 69 amino acids of huntingtin produces typical central nervous system disease in the mouse. It is very unlikely that such a fragment could have retained the function of a protein of over 3000 amino acids [26].

### 3.2. Residues Flanking the PolyQ Affect Its Solubility and Largely Condition Interactions with Heterologous Proteins

It is known that the residues flanking the polyQ influence the solubility of synthetic peptides. Strongly charged flanking residues are effective in maintaining solubility of the peptides with long polyQ stretches [47,76]. The frequency with which COS cells expressing a polyQ of fixed length form inclusions varies according to whether the 17 amino acids *N*-terminal to the polyQ are those of ataxin-2, ataxin-3, huntingtin, or atrophin. Substituting hydrophobic with charged residues in the ataxin-2 flanking sequence greatly reduces aggregate formation [77].

### 3.3. Residues *N*-Terminal to the PolyQ of Huntingtin Promote Aggregation

A number of studies have shown that the sequence *N*-terminal to the polyQ facilitates the aggregation of expanded huntingtin. Most of these studies imply a two-step mechanism by which the *N*-terminal sequence undergoes rapid initial aggregation (but is insufficient to provide stability), followed by the relative slow aggregation of the polyQ into a stable amyloid structure [78].

One of the first reports on the existence of a *cis*-acting activator of polyQ aggregation *N*-terminal to the polyQ of huntingtin came from the study of the chaperonin TRiC. This protein suppresses aggregation of expanded huntingtin by binding to a hydrophobic element *N*-terminal to the polyQ and not by blocking the polyQ itself. The existence of an *N*-terminal amyloidogenic-promoting element explains why huntingtin exon-1 with Q_51_ aggregates much more rapidly than Q_51_ alone. The *N*-terminal element interacts heterotopically with the polyQ and homotopically. The heterotopic interaction promotes the amyloidogenic transformation of the monomer, while the homotypic interaction favors aggregation in a head to head and tail-to-tail fashion [79].

In a variant of this model, the *N*-terminal region, after binding to the polyQ in cis, forms an amphiphilic α-helix with a polar face bound to the polyQ and a nonpolar face free for homotypic interactions. The helical oligomers then promote the formation of amyloid fibrils by increasing the local concentration of the polyQ and by orienting the fibrils in parallel [80]. This model differs from that proposed by Perutz, in 1994, since aggregated polyQ chains are here parallel and not antiparallel as in polar zippers [23].

Thakur and colleagues have also observed that the *N*-terminal sequence promotes aggregation of expanded huntingtin. In their model, expanded polyQ induces the unfolding of the *N*-terminal region and its homotypic aggregation into oligomers. These globular (nonamyloid) oligomers, which are formed without nucleation, consist of a core of *N*-terminal sequences from which the polyQ sequences are excluded. Amyloid nuclei then arise stochastically and trigger rapid amyloid fibrillar growth [81], which no longer requires interaction of the *N*-termini [57]. The formation of oligomers favors fibrillar nucleation as it results in a high local concentration of polyQ. Oligomers that have not undergone nucleation dissociate and release monomers that can then be used for fibrillar extension [82]. Oligomer formation requires a contact limited to the *N*-terminal region since a peptide reduced to the *N*-terminal region will form mixed oligomers with a peptide containing the *N*-terminal region followed by the polyQ [83]. It is worth noting that the *N*-terminal region retains its α-helical structure even after the amyloid fibrils have formed. The *N*-terminal region enhances fibrillar formation without participating in it [57]. None of these studies explain why the rates of aggregation increase with polyQ length and why in nearly all the diseases there is a threshold of about 35–37 glutamines.

The 17 *N*-terminal amino acids of huntingtin may also trigger polyQ aggregation independently of a β-sheet misfolding mechanism. Coiled coil regions are α-helical supersecondary structures that mediate protein-protein interactions and oligomerization. Heptad repeats are the basis of most coiled coils and analysis of huntingtin with Q_72_ has revealed the existence of 12 such heptad repeats within the *N*-terminal region and the polyQ [84]. In this model the 17 *N*-terminal amino acids of huntingtin are a coiled coil-stabilizing element and are, thus, essential. This is in agreement with the findings of Tam and colleagues about the importance of the *N*-terminal sequence for aggregation [79]. The model is at variance with the observations of Thakur and colleagues, according to whom the 17 *N*-terminal residues possess no stable secondary or tertiary structure [81].

### 3.4. Ubiquitination and SUMOylation of the *N*-Terminal Sequence of Huntingtin and Their Influence on PolyQ Aggregation

The *N*-terminal region of huntingtin is the target of modifications that change its propensity to aggregate and its toxicity. There are three lysine residues *N*-terminal to the polyQ, all of which are subject to ubiquitination and SUMOylation. As expected, ubiquitination of the huntingtin exon-1 fragment suppresses cytotoxicity by promoting its degradation [85]. In contrast, the addition of SUMO leads to an increase in neurotoxicity in *Drosophila* and a decrease in the number of inclusions. This effect is direct and is not due to prevention of ubiquitination by SUMOylation. The authors suggest that although SUMOylation decreases inclusion formation, it may increase the amount of toxic oligomers [86].

### 3.5. The *N*-Terminal Sequence of Huntingtin Targets Protein Aggregation to Membranes and Affects Their Integrity

The *N*-terminal sequence of huntingtin contributes to membrane-bound organelle localization of the protein. Mutations in the *N*-terminal region or the deletion of the region lead to loss of membrane association, increased nuclear localization, decreased formation of microscopic aggregates and increased toxicity of expanded huntingtin [87,88]. It has been proposed that oligomerization of expanded huntingtin bound to neuronal membranes alters the physical properties of membranes and cause toxicity, presumably by altering Ca^2+^ concentration in the cytosol [89].

### 3.6. The Polyproline *C*-Terminal to the PolyQ of Huntingtin Inhibits Aggregation

The polyproline (polyP) sequence *C*-terminal to the polyQ of huntingtin decreases both aggregation and toxicity in yeast [79,90,91]. It has been proposed that the polyP had evolved to protect the cell from the toxicity of aggregated polyQ [90,92]. However in normal human huntingtin alleles there is an inverse correlation between polyCAG and polyCCG (encoding polyP) lengths. This linkage disequilibrium is more suggestive of genetic drift than of natural selection [93]. In addition, no polyP adjacent to the polyQ is found in ataxin-1, -3, -6, the androgen receptor or the TATA-binding protein [54,94].

The effect of the polyP on aggregation is directional since polyP added to the *N*-terminal side of the polyQ has no effect on aggregation. This is presumably because a proline residue *C*-terminal to a glutamine residue suppresses much of the conformational freedom of the glutamine and favors its folding into a compact structure incompatible with β-sheet formation [95,96]. Molecular dynamics simulations show that the polyP of the exon-1 encoded huntingtin fragment forms type II helices and prevents the polyQ from forming β-sheets [97]. Proline is known to be a β-breaker and therefore is structurally incompatible with β-sheet formation [98]. It has been suggested that the presence of a polyP is the reason for which the threshold of huntingtin aggregation (about Q_35_) is greater than that of pure polyQ peptides (Q_6_) [92]. However although ataxin-1, -3, the androgen receptor or the TATA-binding protein does not possess a polyP adjacent to the polyQ, they aggregate when polyQ length exceeds a value of about 35 residues.

It has been proposed that the polyQ of huntingtin is normally a flexible domain whose flexibility permits the folding back of the 17 *N*-terminal amino acids towards the polyP. Such a conformation requires the interacting protein PACSIN1. The folding back is reduced when the polyQ exceeds Q_37_, the pathological threshold for Huntington disease [99]. How the altered conformation of huntingtin becomes toxic for neurons is not clear.

## 4. Aggregates of Expanded Huntingtin Consist of *N*-Terminal Fragments of the Protein

### 4.1. Evidence of the Presence of Fragmented Huntingtin in Inclusions

The fact that the inclusions present in the brain of patients with Huntington disease are stained by antibodies directed against residues located in the vicinity of the polyQ, but not by antibodies directed against more *C*-terminal parts of huntingtin, suggested very early that inclusions were composed of *N*-terminal fragments of the expanded protein [21,100]. When the inclusions found in the brain of patients with Huntington disease are incubated in pure formic acid, they release multiple *N*-terminal fragments of expanded huntingtin as well as oligomers and polymers of the protein. The expanded huntingtin fragments are resolved by electrophoresis as a smear of bands immunoreactive for the 1C2 antibody, which specifically stains the expanded polyQ. Since the molecular weights of the fragments range from 50 to 150 kDa, these fragments are predicted to contain between about 350 and 1300 residues. This is corroborated by the fact that the 4C8 antibody (which recognizes an epitope located between residues 490 and 580 of huntingtin) does not stain the smaller fragments present within the 1C2-stained smear. The fragments have a similar range of sizes whether they are derived from cortex or caudate-putamen, or from adult or juvenile patients [20,38]. Presence of aggregated *N*-terminal huntingtin fragments in nuclei has also been reported in cultured cells expressing expanded intact huntingtin [101] and in Huntington disease brain [38,39].

### 4.2. Aggregating Properties and Toxicity of Fragments

The importance of the *N*-terminal fragments of expanded huntingtin in the pathophysiology of the disease has been lent support by the fact that they aggregate much more rapidly and are much more toxic than the unfragmented protein, whether in cultured cells, transgenic mice, or monkeys [27,102,103,104]. Recent evidence obtained from induced pluripotent stem cells (iPS) has provided additional support as to the importance of the *N*-terminal fragments of huntingtin. Neuronally differentiated iPS cells derived from transgenic animals expressing huntingtin exon-1 with expanded polyQ form inclusions [105,106], whereas fibroblasts of patients with Huntington disease do not form microscopic aggregates [107,108,109].

Proteolytic fragmentation of ataxin-3 has also been reported. Neurons derived from iPS cells of patients with SCA3 form SDS-insoluble aggregates only if they have been excited with l-glutamate. The glutamate-induced aggregation requires the Ca^2+^-dependent proteolysis of ataxin-3 [110]. Huntington disease-iPS neurons transplanted into normal mouse brain start forming inclusions about 30 weeks after they have been transplanted. This long delay might correspond to the time necessary to proteolyze huntingtin in fragments that will then aggregate [111].

### 4.3. Proteolytic Enzymes that Generate the Fragments

Endoproteases such as caspases-3 and -6, calpain, and aspartic endopeptidases participate in the proteolysis of huntingtin [101,112,113,114,115,116]. Prevention of caspase-6 mediated cleavage of huntingtin has been shown to alleviate Huntington disease pathology in YAC transgenic mice [117]. The smallest *N*-terminal fragment of huntingtin observed in knockin mice might result from CAG repeat length-dependent splicing of the huntingtin transcript, rather than proteolysis [118]. However, in view of the multitude of fragments that must form the smear of immunoreactive huntingtin observed in knockin mice and in Huntington disease brain, it seems more likely that these fragments stem from the action of multiple proteolytic activities with little specificity [20]. It is possible that a discrete fragment generated by a single endoprotease is subject to further proteolysis with little specificity, giving to the initial endoprotease an essential role in the pathology. This may be the case for calpain in SCA3 [110].

## 5. Microscopic Aggregates: Inclusions and Fibrils

### 5.1. Diversity in Morphology, Subcellular Distribution and Number of Inclusions

The inclusions found in the brain of R6/2 mice vary in morphology, subcellular localization, and proteasome subunit composition [52]. In Huntington disease, inclusions vary by their morphology and by their nuclear or cytoplasmic localization. In both juvenile and adult Huntington disease, nuclear inclusions possess a uniform volume of about 4 μm^3^. In contrast, cytoplasmic inclusions are highly variable in size in both juvenile (1–40 μm^3^) and adult patients (1–150 μm^3^). The volume of some cytoplasmic inclusions reaches approximately 300 μm^3^ in adult patients. Nuclear inclusions are about twenty times more abundant in juvenile than in adult patients. This is not only because the overall number of inclusions is greater in juvenile cases but also because inclusions are predominantly nuclear in juvenile cases and cytoplasmic in adult cases [20].

### 5.2. Diversity of Protein Secondary Structure in Microscopic Aggregates

Heterogeneity of amyloid aggregates was first demonstrated by Nekooki-Machida and collaborators. These authors showed by FTIR analysis that prion-like amplified huntingtin aggregates adopt different structures depending on the temperature at which they form. Amyloid that have formed at 4 °C contain loop/turn structures (absorbance peak at 1655–1680 cm^−1^) and β-sheets (1640 cm^−1^), whereas amyloids formed at 37 °C contain extended β-sheets (1615 cm^−1^) in addition to the classical β-sheets at 1640 cm^−1^ [119]. Using synchrotron FTIR microspectroscopy, we have recently discovered that the proteins composing the inclusions present in the brain of patients with Huntington disease possess polymorphic secondary structures. Aggregated proteins can adopt two kinds of amyloid conformations or be amorphous, depending on the length of the polyQ, the subcellular location of the inclusions (nuclear or cytoplasmic) and the brain region [60].

### 5.3. Toxicity of Microscopic Aggregates

Considerable evidence supports the idea that huntingtin aggregation causes neuronal death in Huntington disease. For instance, the frequency of cortical inclusions in neurons is directly correlated with the severity of the disease [100,120], and inclusion formation precedes the onset of symptoms in transgenic mice and in human brain [27,121]. Aggregates of polyQ formed *in vitro* and introduced into cultured cells produce cell death [122]. It has been shown that suppression or reduction of aggregate formation is correlated with suppression or alleviation of toxicity both in transgenic animals and in cell culture. It has been argued that inclusions are not the cause of cell lethality and may even be protective. By various means, these investigators have found absence of correlation between the presence of inclusions and cell death [123,124]. However, all this evidence applies only to certain kinds of microscopically visible aggregates and does not preclude that either microscopic aggregates formed under different conditions or submicroscopic aggregates such as oligomers might be toxic.

### 5.4. Influence of Protein Structure on Toxicity of Microscopic Aggregates

When dealing with toxicity, it is important to take into consideration the morphological, biochemical and structural heterogeneity of microscopic aggregates. The following examples demonstrate the influence of these parameters on toxicity of aggregates. In *Drosophila* expressing huntingtin exon-1 with Q_53_, the chaperone Hsp70 and its cochaperone Hsp40 interact with the expanded fragment, thus delaying the formation of detergent-insoluble aggregates and promoting instead the formation of detergent-soluble aggregates. As in these flies, Hsp70 and Hsp40 are neuroprotective, it is thought that detergent-insoluble aggregates are toxic whereas soluble ones are not [125]. Mice expressing huntingtin exon-1 with very long repeats of up to 400 CAGs develop inclusions that differ in size and shape from those of the R6/2 line and tend to be cytoplasmic rather than nuclear as they are in R6/2 mice. It is paradoxical that in these mice, which possess 400 CAGs, disease should develop much later than in the R6/2 mice in which CAG length is only about 130 repeats [126]. Therefore, morphology, biochemical properties, and nuclear or cytoplasmic localization are associated with variable rates of neuronal death. This may reflect differences in protein structure between different populations of inclusions.

It has been demonstrated that the amyloid conformation of aggregates in itself can condition toxicity. Fibrillar aggregates found in the brain of R6/2 mice possess an amyloid structure whose conformation and toxicity vary according to the cerebral region where they form. Nekooki-Machida and colleagues have used purified SDS-resistant aggregates of R6/2 mice as seeds for the aggregation of the huntingtin with Q_42_. By this method, the aggregates are replicated in a prion-like manner while presumably maintaining their original structure. Structural analysis of the amplified aggregates was carried out by FTIR spectroscopy. The amyloid aggregates of striatum and cortex were found to be structurally different from those of cerebellum and hippocampus. Striatal and cortical amyloids possessed flexible and thermally labile loop/turn structures (1655–1680 cm^−1^) in addition to the mostly β-sheet structures (1640 cm^−1^). Amyloid fibrils amplified from either cerebellum or hippocampus showed higher resistance to heat treatment, and possessed more extended and buried β-sheets (more intermolecular β-sheets at 1615 cm^−1^) in addition to the mostly β-sheet structures. The cytotoxicity of amyloids was then assessed in huntingtin Q_150_-GFP neuro2a cells. Amyloids formed from striatal seeds were found to be more toxic to neuro2a cells than those amplified from either hippocampus or cerebellum. It was concluded that the toxicity of striatal amyloid aggregates was caused by the presence of exposed polyQs, capable of deleterious interactions with important cellular proteins. In contrast, the limited dynamics of a polyQ buried into an amyloid core would not lead to interactions with cellular proteins [119].

Another example of the influence of structure on toxicity is given by the age-dependent toxicity of aggregates produced in *Drosophila*. Amyloid aggregates formed in older flies are less resistant to SDS, are more effective at seeding polymerization and are associated with a more severe disease than those formed in younger *Drosophila*. This implies that some structural characteristics of the aggregates govern their toxicity [68]. These findings imply that SDS-resistant aggregates are less toxic than SDS-soluble ones and therefore are in contradiction with those of Muchowski and colleagues, although both studies were carried out in *Drosophila* [125].

Heterogeneity of the aggregates and its relevance to toxicity has also been illustrated in patients with Huntington disease. We have shown by infrared microspectroscopy that amyloid inclusions enriched in both β-sheets (1627 cm^−1^) and β-sheets/unordered structures (1639 cm^−1^) are characteristic of the brain regions most affected by Huntington disease and therefore are likely to be particularly toxic to neurons. In contrast, amyloid inclusions enriched only in β-sheet structures (1627 cm^−1^) are specific to less affected brain regions and may therefore be less toxic [60].

Thakur and Wetzel have addressed the question of the toxicity of the polyQ secondary structure by mutagenesis. They have induced β-turns in polyQ peptides by introducing Pro-Gly replacements at different intervals. A peptide consisting of four Q_9_ or Q_10_ repeats linked by Pro-Gly pairs aggregates as efficiently as an uninterrupted Q_45_. The authors propose a model in which polyQ forms alternative β-strands and β-turns with Q_7_ or Q_8_ in each β-strand [127]. It was later found that the Pro-Gly polyQ peptides form aggregates in neuronal cell lines and cause toxicity. These data suggest the importance of a compact β-rich hairpin structure in the toxicity of huntingtin [128,129].

## 6. Oligomers

### 6.1. Discovery

Huntingtin oligomers have been discovered belatedly compared to fibrils and inclusions. The first report on oligomers was published by the group of Ross and colleagues [130], using a huntingtin exon-1 protein with Q_44_ fused to the maltose-binding protein. They observed by electron microscopy the generation of globular oligomeric assemblies as early as 30 min after the cleavage of the carrier protein. Upon examination by electron and atomic force microscopy, these soluble intermediate-sized aggregates were found to be 4 to 10 nm in diameter. Oligomers were later found in cells [131] and in mice simulating SBMA [132]. By electron microscopy, huntingtin inclusions produced by transiently transfected MCF7 cells or found in the brain of patients with Huntington disease show globular aggregates concentrated at the periphery or in the center of the inclusions, respectively [133]. Thus, the oligomers are components and possibly precursors of the inclusions.

### 6.2. Recently Revisited Tenets on the Formation of Oligomers

While simple polyQ peptides are classically thought to undergo nucleated growth polymerization with direct formation of amyloid-like aggregates [47,134,135], aggregation products of huntingtin exon-1 include, in addition to amyloid fibrils [28], oligomers, and protofibrils [130] that many feel are more relevant to disease development [136]. A recent work has demonstrated the formation of oligomers by what are essentially homopolymers of polyQ. Oligomeric intermediates had been previously missed possibly because of their ephemeral nature due to the very rapid formation and accretion of polyQ homopolymers into fibrillar aggregates [133].

It has long been postulated that oligomers consist of expanded polyQ without the participation of normal-length polyQ. Study by atomic force microscopy of the aggregation of GST-tagged huntingtin fragments with polyQs of variable lengths led Muchowski and coworkers to notice that even a normal-length Q_20_-bearing fusion protein aggregates into oligomers, but is unable to produce fibrils [133]. It has also been shown that full length ataxin-3 carrying Q_24_ forms oligomers but not fibrils [137]. Although even *N*-terminal fragments of huntingtin with very short polyQs (< Q8) can assemble into oligomers [82], the kinetics of polyQ oligomerization and nucleation are much faster for longer polyQs. It is worth noting that the presence of soluble oligomers of normal huntingtin *in vivo* has never been reported.

### 6.3. Oligomers Precede Microscopic Aggregates

*In vitro,* huntingtin exon-1 polyQ oligomers precede fibrillar aggregates and can be their direct precursors [130,133]. In primary striatal neurons transduced with a lentivirus encoding the huntingtin exon-1 protein containing Q_72_, oligomers are detected four days after infection, *i.e.*, three days before the appearance of inclusions [138]. Likewise, oligomers containing truncated atrophin-1 with Q_56_ have been shown to assemble in COS-7 cells and to precede the formation of inclusions [59]. Oligomers are detected in presymptomatic mice far before the emergence of the inclusions. In mice expressing the full-length human androgen receptor cDNA containing 112 CAGs, oligomers are detected 52 days after birth, whereas motor symptoms start developing 75 days after birth and inclusions become visible after 150 days [132]. Lotz and colleagues have used agarose gel electrophoresis and size exclusion chromatography followed by FRET to study the kinetics of oligomer formation in knockin mice that express a human huntingtin with Q_150_. Water-soluble oligomers were detected at one month of age long before pathology was observed. In the knockin mouse brain, the pool size of soluble oligomers declined with age until about eight months, from which time onwards the pool-size seemed to remain at steady state. The decline of the soluble oligomer pool inversely correlated with the formation and accumulation of insoluble aggregates containing expanded huntingtin. Thus in these mice, insoluble but not soluble oligomers were correlated with the appearance of the disease [139].

### 6.4. Heterogeneity of Oligomers

Soon after the discovery of oligomers, it became apparent that oligomeric polyQ is heterogeneous. Oligomers with spherical or annular morphologies are formed by expanded huntingtin fragments *in vitro* [140]. Anti-huntingtin antibodies differentially recognize huntingtin oligomers formed *in vitro*, presumably because the affinity of the antibodies depends on the conformation of the oligomerized protein [141]. An antibody specific for the conformation of oligomeric amyloid-prone proteins has affinity for a subset of polyQ species *in vivo* [59,142]. In neuro2a cells expressing the huntingtin exon-1 protein with Q_46_, two populations of oligomers with sedimentation velocities of 50 and 140 S are identified by fluorescence-adapted sedimentation velocity. These oligomers are predicted to be comprised of about 80 and 200 molecules, respectively [143].

The fact that oligomers are resolved as smears of immunoreactive bands by electrophoresis also suggests the existence of a complex oligomeric population. Using agarose gel electrophoresis, a smear of immunoreactive oligomers with an apparent molecular weight greater than 650 kDa is detected in HN10 neuroblastoma and striatal primary cells producing the huntingtin exon-1 protein with Q_72_. No oligomers are detected in cells synthesizing the protein with Q_25_ [138]. The fact that smears comparable to those observed in cultured cells are present in the brains of R6/2 and HdhQ150 mice shows that the oligomers of cultured cells and mice are very similar. Agarose gel analysis of the oligomers of R6/2 brain reveals that cytoplasmic oligomers are initially smaller than nuclear ones, but as the disease progresses, cytoplasmic oligomers increase greatly in size, while the size of nuclear oligomers remains unchanged. This shows that oligomers are dynamic structures influenced by the cellular context. In mice expressing the full-length human androgen receptor cDNA with 112 CAGs, oligomers are resolved as a smear running at ~250–450 kDa by denaturing polyacrylamide gel electrophoresis [132]. By non-denaturing electrophoresis huntingtin oligomers migrate as 1100-kDa particles [144].

When proteins extracted from the brain of patients with Huntington disease are analyzed by Western blotting, monomeric expanded huntingtin is replaced by a smear extending above the expected position of the monomer [19,145,146]. We have proposed that this smear corresponds to oligomers of huntingtin, which are resistant to the SDS used in the loading buffer [38]. In juvenile patients, the oligomers exist in a water-soluble form in the cytosol but are part of larger aggregates in the nuclei (presumably the inclusions) since their release from nuclear preparation requires incubation in formic acid [38]. The presence of oligomers in the inclusions has also been demonstrated by formic acid treatment of inclusions purified from the brain of patients [20]. It is curious that the oligomers themselves are resistant to formic acid. This has suggested that the oligomers were crosslinked by covalent bonds, such as those formed by transglutaminase [38,76,147]. As a broad range of *N*-terminal fragments of expanded huntingtin is present in the brain of patients with Huntington disease, fragments may aggregate into different kinds of oligomers and, thus, generate a heterogeneous population of oligomers.

### 6.5. Secondary Structure of the Oligomerized Proteins

There is little data on the structure of oligomeric polyQ, presumably because of its transitory nature and the difficulty of its isolation from brain. Huntingtin oligomers formed *in vitro* and analyzed by infrared spectroscopy produce two minor bands in the 1620–1640 cm^−1^ range. Both bands indicate either a β-sheet or β-turn structure, but not necessarily an amyloid β-sheet structure. This is in contrast to the huntingtin fibrils in which strong increase in absorbance at 1620 cm^−1^ is characteristic of an amyloid structure [130]. Lack of a clear assignment of oligomers to an amyloid structure has also been recently reported [81]. The true amyloid nature of the fibrils but not of the oligomers may explain why Congo red does not interfere with the formation of oligomers but prevents fibril formation [130].

As mentioned above, the first 17 amino acids of huntingtin, whether alone or flanked by a polyQ, have been shown to build up oligomeric assemblies enriched in α-helix [81,82]. In these assemblies, the 17 *N*-terminal residues of huntingtin constitute the core whereas the polyQ is exposed and unstructured. Such assemblies would be consistent with the fact that, in Western blot analyses, monoclonal antibodies directed against the polyQ (1C2, MW1, and 3B5H10) stain the oligomers, whereas anti-*N*-terminal huntingtin antibodies do not [20,144,148].

In contrast to the *in vitro* data, the little *in vivo* data available support a β-sheet arrangement of oligomers. In cultured cells, investigation of the architecture of truncated atrophin-1 oligomers using FRET indicates a parallel β-sheet or head-to-tail cylindrical β-sheet conformation [59]. Evidence obtained by synchrotron-based FTIR suggests that oligomers found in the brain of patients with Huntington disease are enriched in antiparallel β-sheets (absorbance peaks at 1627 and 1693 cm^−1^) and in probably unordered structures (1639 cm^−1^). As these oligomers are cytoplasmic in adult HD and nuclear in juvenile HD case, their subcellular localization corresponds to that of the inclusions. Therefore the oligomers might be the precursors of the inclusions [60].

### 6.6. Toxicity of Oligomers

Oligomers of expanded polyQ are generally considered to be more toxic than either fibrils or inclusions. Using FRET confocal microscopy, it is possible to distinguish in cultured cells oligomeric polyQ from monomers and inclusions. Neuroblastoma cells stably expressing expanded polyQ and containing oligomers die faster than those with either monomers or inclusions alone [59]. Shortstop mice contain exons 1 and 2 of human huntingtin with 120 CAGs. They express an *N*-terminal fragment of huntingtin, which is 27-residues longer than that of the R6/2 mice. In contrast to the R6/2 mice, the shortstop mice do not show evidence of neurological disease [149]. When the exon-1 encoded huntingtin with Q_44_ is produced in cultured cells, a transient oligomeric species is resolved by nondenaturing polyacrylamide gel electrophoresis. No such oligomeric species is detected when the shortstop protein with a polyQ of the same length is produced. It is concluded that the transient oligomer might be the toxic species in the R6/2 mice. It is not clear why the shortstop protein does not form such an oligomer [144]. Castration of mice modeling SBMA arrests the disease within three weeks and causes the concomitant disappearance of oligomers [132]. Feeding Huntington disease transgenic flies with polyphenol epigallocatechin-gallate alleviates photoreceptor degeneration and motor impairment, presumably by shifting the balance of aggregate formation in favor of oligomers to the detriment of large aggregates [150]. In transgenic *Drosophila* that initiate synthesis of an ataxin-3 with Q_78_ when their temperature is increased to 25 °C, there is progressive accumulation of SDS-insoluble aggregates of polyQ with varying sizes. The Hsc70 molecular chaperone does not affect neurotoxicity. As Hsc70 prevents the accumulation of large aggregates but does not affect polyQ oligomers, it is concluded that the latter constitute the toxic species [151].

In summary, unstable, heterogeneous pre-fibrillar aggregates are widely acknowledged to be responsible for amyloid toxicity, while mature fibrils are considered stable, harmless reservoirs of toxic species [152]. However, it is worth noting that in one report fibrils were found to be more toxic than oligomers. These authors reached different conclusions from most other investigators probably because of the way by which they normalized the concentrations of fibrils and oligomers. In most studies, fibrils and oligomers are normalized by monomer concentration. Since fibrils are much larger than oligomers, the number of oligomeric particles to which the cells are exposed is much greater than that of fibrils. Melki and colleagues used instead a normalization by concentration of particles, whether fibrils or oligomers. As a consequence, the concentration of fibrils used was thousands of time higher than in other studies and fibrils were found to be more toxic than oligomers. Fibril toxicity was attributed to their ability to bind and permeabilize cell membranes [153].

## 7. Monomers

### 7.1. Water-Soluble Fragments of Monomeric Huntingtin in Affected Brain Regions

As the expanded allele in Huntington disease is dominant, patients are nearly always heterozygous and therefore possess both normal and expanded huntingtin. Normal huntingtin is found in monomeric form in both affected and unaffected brain regions. In contrast, expanded huntingtin is found in a monomeric and intact form in relatively spared brain regions, such as the cerebellum and the hippocampus, whereas it is replaced by monomeric *N*-terminal fragments, oligomers, and microscopic aggregates (inclusions) in affected regions, such as the cortex and the caudate/striatum.

*N*-terminal fragments of both normal and expanded huntingtin are generated in brain [154]. Although these fragments must be generated in both affected and unaffected brain regions, water-soluble *N*-terminal fragments of expanded huntingtin are found in Huntington disease cortex but not in the cerebellum [20]. These fragments tend to be larger than those composing the inclusions. It is likely that shorter fragments aggregate immediately after they are generated, while longer fragments can remain in a water-soluble form.

Fourteen different *N*-terminal fragments of expanded huntingtin are detected in a knockin mouse that synthesizes human huntingtin with Q_150_. The fragments are resolved as discrete bands in young and adult mice, but they form a continuous smear in older animals [154]. The smear of fragments of older animals is very similar to that found in the cortex of patients with Huntington disease [20]. The soluble huntingtin fragments of knockin mouse neurons are initially cytoplasmic but as the mouse ages, the fragments disappear from the cytoplasm and accumulate in an insoluble form in nuclei [154]. It remains to be determined whether the water-soluble fragments containing the expanded polyQ are toxic to neurons.

### 7.2. Secondary Structure of Monomeric Polyglutamine

The conformation of oligoglutamines has been predicted to be random coil by computer simulation [155]. Similar conclusions have been drawn from the study of small polyQs in aqueous solution, either flanked by charged residues and analyzed by circular dichroism [156] or solubilized by fusion with glutathione S-transferase and investigated by infrared and nuclear magnetic resonance spectroscopy [157,158]. More recently, the crystal structure of the huntingtin-exon 1 fragment with a normal-length polyQ has been determined [159]. The polyQ was found to exist in multiple conformations likely in equilibrium: α-helix, random coil and extended loop. It was proposed that this equilibrium was modulated by several factors like the length of the polyQ, interactions with protein partners, temperature and ionic composition. Each of the multiple conformations may be transient and thus no single conformation may predominate. This would explain why biophysical techniques delivering a global structural overview have attributed a random coil structure to polyQ. PolyQs maintained in solution by fusion to GST have been shown to possess a random coil conformation whether their length is below or above the pathological threshold [157,158]. Thus, both long and short polyQs appear to be disordered in aqueous solution.

Another structural feature of polyQs in solution is their compactness, as shown by molecular dynamics simulations [160], fluorescence correlation spectroscopy [161], single-molecule force-clamp technique [162], and small-angle X-ray crystallography [163]. The average compactness and the amplitude of conformational fluctuations increase with the length of the polyQ [160].

Important information on the structure of expanded polyQ in monomeric huntingtin has been obtained using the so-called conformational antibodies, which are assumed to recognize specific conformations adopted by the polyQ domain. Determination by X-ray crystallography of the crystal structure of polyQ bound to the MW1 and 1C2 antibodies has demonstrated that the polyQ can adopt a linear, extended and unstructured configuration, consistent with the “linear lattice” model [132,145,158,163,164]. The two antibodies bind with greater affinity the expanded form of the protein because more copies of the epitope are present in expanded than in normal huntingtin and not because the expanded polyQ adopts a specific conformation.

This is in contrast to data inferred from the co-crystallization of the polyQ domain with another conformational antibody, designated 3B5H10. This antibody was thought to recognize a compact two-stranded β-hairpin structure specifically adopted by expanded polyQ [163]. However, Trottier and colleagues have challenged this conclusion by providing evidence that 3B5H10 binds a short polyQ epitope similar to that recognized by the MW1 and 1C2 antibodies [165]. 

The fact that different antibodies directed against the polyQ each recognize a different set of expanded huntingtin exon-1 fragments lends support to the idea that each of these sets has a different structure [141]. This would be consistent with the X-ray crystallography analyses that have shown that *N*-terminal fragments of huntigtin can adopt varying structures [159].

The general conclusion is that the polyQ in monomeric huntingtin is conformationally disordered and compact whether its length is normal or excessive. The polyQ may also transiently adopt regular secondary structures. The main difference between normal and expanded polyQ is the faster aggregation of the latter [158]. Such results are in contradiction with previous experiments supporting the idea that the threshold of polyQ length-dependent toxicity results from the unique structural properties acquired by expanded polyQ [28].

### 7.3. Toxicity of Monomeric Polyglutamine

Aggregated polyQ is generally thought to cause neurological disease while the monomeric protein is considered as benign. However, several studies have challenged this scenario, and have purported to demonstrate the neurotoxicity of monomeric proteins bearing an abnormally long polyQ. Nagai and coworkers have observed that the structure of a thio-Q_62_ monomer can shift from mostly α-helical to enriched in β-sheets, thus prefacing its assembly into amyloid fibrils. That misfolding could occur at the level of the monomer goes against the generally accepted view that misfolding is concomitant with aggregation. Microinjection of the thio-Q_62_ monomer with the β-sheet structure in COS cells causes rapid and extensive cell death [166]. In these experiments, it is assumed that most of injected protein remains monomeric since it does not react with an anti-Aβ antibody specific of amyloid oligomers in general [142]. This suggests that the toxicity of the polyQ monomer is due to its misfolding into a β-sheet conformation. 

Through the use of a panel of anti-huntingtin antibodies and the tracking of thousands of neurons with an automated microscope, Finkbeiner and coworkers have sought to identify pathogenic variants of expanded huntingtin. They have found a correlation between 3B5H10 antibody staining and neuronal death. As the 3B5H10 antibody was thought to be specific to a compact β-hairpin structure, it was concluded that this structure was particularly neurotoxic. Compact β-hairpins are found in monomers and possibly very small oligomers of huntingtin, but are absent from larger oligomers and higher order aggregates [167]. Similar conclusions have been drawn by Zhang and coworkers. They have demonstrated that expanded huntingtin fragments designed to display a compact β-structure are toxic to cultured neuro2a cells in proportion to their reactivity to the 3B5H10 antibody [128]. The fact that huntingtin bearing a compact β-structure has deleterious effects without forming microscopic aggregates emphasizes the toxicity of monomers and small oligomers of huntingtin, providing that they possess this compact β-conformation. We have seen, however, that the conformational nature of the epitope recognized by the 3B5H10 antibody has been challenged [165].

To summarize, huntingtin monomers, whether disordered or structured, might be toxic and thus might constitute interesting therapeutic targets. Their damaging properties may be explained by an increased accessibility of the polyQ, which would facilitate abnormal interactions with other cellular components. Consistent with this hypothesis, the polyQ region of soluble monomeric expanded huntingtin fragments has been shown to interact with the polyQ of the TATA-box binding protein, and to result in the loss of function of the latter [168].

## 8. Conclusions

**Figure 1:**
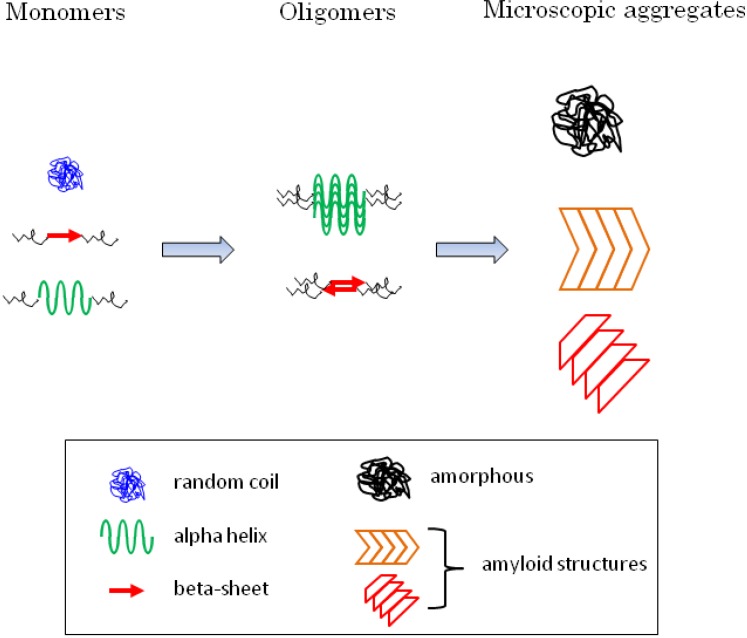
Monomeric and aggregated expanded huntingtin consist of structurally heterogeneous particles. The polyQ in soluble monomeric fragments of expanded huntingtin generally adopts a random coil structure. It may also display regular structures such as α-helix or β-sheet. Oligomers may be made up of an α-helix core formed by the first seventeen amino-acids of huntingtin, with the polyQ being exposed and unstructured. Alternatively they may consist of small aggregates with buried polyQ, presumably with an antiparallel β-sheet rich structure. Microscopic aggregates (inclusions), composed of expanded huntingtin and other sequestered proteins, include amorphous aggregates and polymorphic amyloid conglomerates rich in β-sheets with antiparallel and/or parallel arrangements. Monomers, oligomers, and microscopic aggregates of expanded huntingtin, therefore, each comprise heterogeneous populations in terms of structure. They are additionally heterogeneous in size and morphology (see text).

PolyQ aggregation is likely to play a major role in Huntington disease and other polyQ diseases. Ever since the discovery of inclusions, the research community has been divided about their toxicity. Recent data has suggested that oligomers and possibly monomers of expanded polyQ are harmful to cells. The controversy on the toxicity of inclusions springs from contradictory data obtained on a variety of experimental systems. The discrepancies may be reconciled by taking into account the diversity of aggregated and nonaggregated forms of the expanded proteins (Figure 1).

As illustrated in this review, morphology, composition and structure of polyQ aggregates vary according to experimental conditions, subcellular localization, tissue environment and length of the polyQ [60,120]. For instance post-translational modifications or proteolysis of huntingtin may differ depending on the brain region or the neuronal cell compartments, giving rise to distinct fragments of huntingtin, which would be prone to adopt variable secondary structures and thereby produce structurally different aggregates. It remains to be understood how polyQ species with different secondary structures, whether they are aggregated or monomeric, cause neuronal death.

Several hypotheses have been proposed to explain aggregate toxicity. Sequestration of heterologous proteins within the aggregates may render these proteins unable to exert their function. Proteins rich in Q/N residues, such as the TATA-box binding protein, the cAMP-response element-binding protein, TIA-1 or normal huntingtin are trapped into inclusions [66,144,168,169,170]. The structure of the polyQ aggregates may condition their affinity for heterologous proteins as well as the nature of the sequestered proteins. Due to its adhesive properties, a polyQ exposed on the surface of the aggregate may readily sequester heterologous proteins in the aggregates. This would not be the case when the polyQ is buried inside the aggregate. It has also been proposed that polyQ oligomers penetrate cell membranes and form toxic ion-conducting channels that depolarize membranes [25,171,172,173]. In this case, it is possible that the structure of polyQ aggregates influences their ability to insert into membranes. Further investigations are necessary to understand the precise mechanisms by which the structure of the expanded polyQ, whether in an aggregated or monomeric state, modulates toxicity.

## Abbreviations

DRPLA: dentatorubral-pallidoluysian atrophy
FTIR spectroscopy: Fourier-transform infrared spectroscopy
FRET: fluorescence resonance energy transfer
GST: glutathione S-transferase
PolyQ: polyglutamine
SBMA: spinobulbar muscular atrophy
SCA: spinocerebellar ataxia
TIA-1: T-cell intracellular antigen-1
